# Assessment of left ventricular volumes using simplified 3-D echocardiography and computed tomography – a phantom and clinical study

**DOI:** 10.1186/1476-7120-6-26

**Published:** 2008-06-04

**Authors:** Mattias Mårtensson, Reidar Winter, Kerstin Cederlund, Jonaz Ripsweden, Habib Mir-Akbari, Jacek Nowak, Lars-Åke Brodin

**Affiliations:** 1 School for Technology and Health, Royal Institute of Technology, Flemingsberg, Stockholm, Sweden; 2 Department of Clinical Physiology, Karolinska University Hospital in Huddinge, Stockholm, Sweden; 3 Department of Radiology, Karolinska University Hospital in Huddinge, Stockholm, Sweden; 4 Department of Cardiology, Karolinska University Hospital in Huddinge, Stockholm, Sweden

## Abstract

**Objectives:**

To compare the accuracy of simplified 3-dimensional (3-D) echocardiography vs. multi-slice computed tomography (MSCT) software for the quantification of left ventricular (LV) volumes.

**Design:**

Three-D echocardiography (3-planes approach) and MSCT-CardIQ software were calibrated by measuring known volumes of 10 phantoms designed to closely mimic blood-endocardium interface. Subsequently, LV volumes were measured with both the methods in 9 patients referred routinely for coronary angiography and the agreement between the measurements was evaluated.

**Results:**

Simplified 3D-echocardiography provided higher degree of agreement between the measured and true phantom volumes (mean difference 0 ± 1 ml, variation range +4 to -4 ml) than MSCT software (mean difference 6 ± 5 ml; variation range +22 to -10 ml). The agreement between LV measurements in the patients was considerably poorer, with significantly larger volumes produced by MSCT (mean difference -23 ± 40 ml, variation between +93 and -138 ml).

**Conclusion:**

Simplified 3-D echocardiography provides more accurate assessment of phantom volumes than MSCT-CardIQ software. The discrepancy between the results of LV measurements with the two methods is even greater and does not warrant their interchangeable diagnostic use.

## Background

Echocardiography and x-ray based computed tomography provides possibilities of detailed evaluation of cardiac morphology and function and the introduction of these techniques constitutes without any doubt an important landmark in the history of diagnostic cardiology. Today, both the methods not only form a backbone of diagnostic cardiac procedures but also are much appreciated as sensitive research instruments. Recently, the diagnostic capacity of echocardiography has been further improved by the addition of 3-dimensional cardiac imaging.

An important link in the process of cardiac diagnostics is accurate estimation of left ventricular volume. In this respect, MSCT has been shown to be both feasible and accurate when compared with left ventriculography [1], magnetic resonance techniques [2-4], and 2-dimensional echocardiography [4-7]. At the same time, the diagnostic performance of 3-D echocardiography has been demonstrated to be superior to that provided by 2-dimensional echocardiographic imaging [8-13]and a strong correlation was observed between the results obtained with 3-D echocardiographic technique and magnetic resonance imaging [9,11,14-17].

However, the results of a recently published study indicate that even though both 3-D echocardiography and MSCT measurements correlate highly with magnetic resonance imaging, 3-D echocardiography compares more favourably in this respect than MSCT that tend to overestimate the magnetic resonance values [17]. Therefore, the aim of this study was to evaluate further the accuracy of both methods in the assessment of LV volumes in clinical subjects and to verify the results of volume measurements in *in vitro *setting using phantoms.

## Methods

### Phantoms

Ten different cone-shaped phantoms were used in the study. Eight of them were manufactured with a symmetric geometric structure and two with asymmetric geometry that was considered to mimic LV with an aneurysm. All the phantoms were manufactured using water-based microbiology agar (1:30, agar:water, w/w) described by Burlew et al. [18]. In order to mimic blood-myocardium interface, a glass powder with very fine granularity was added to the outer compartment of the phantom considered to represent the cardiac walls (1:65, glass powder:water-based agar, w/w), thus increasing the echocardiographic density. On the other hand, iodixanol (Visipaque, GE Healthcare, Little Chalfont, UK; 320 mg I/ml) was added to the inner compartment of the phantom representing intracavital blood volume (1:20; iodixanol:waterbased agar, w/w) in order to increase the x-ray attenuation. The chosen concentration of iodixanol resulted in approximately the same Hounsfield values as in normal clinical scans. The specific weight of the mixture for the inner compartment was measured and the phantoms were weighed before and after adding the mixture. The exact volumes of the inner compartment of the phantoms (called phantom volumes) could then be calculated and ranged between 39 and 334 mL (Table 1).

**Table 1:** True phantom volumes and the volumes obtained with simplified 3-D echocardiography and MSCT-CardIQ software.

**Phantoms** *(number)*	**True volume** *(ml)*	**Measured volume** *(ml)*	
		
		**3-D echo-cardiography**	**MSCT-CardIQ software**
		*(mean ± SD; n = 5)*	*(mean ± SD; n = 5)*
1	39	36.6 ± 0.55	40.4 ± 1.14
2	98	99.4 ± 0.55	99.8 ± 1.10
3	334	333.8 ± 0.84	348.4 ± 5.64
4	293	291.6 ± 1.14	296.6 ± 0.55
5	127	128.0 ± 1.23	131.6 ± 1.68
6	52	51.6 ± 0.55	54.6 ± 1.52
7	202	202.2 ± 0.84	209.2 ± 1.64
8	246	246.0 ± 1.23	251.8 ± 1.48
9	68	68.0 ± 1.30	72.6 ± 0.55
10	157	158.6 ± 3.44	172.6 ± 0.55

(1 – 7: symmetric, and 9 – 10: asymmetric phantoms)

The speed of ultrasound in the phantom material was measured in two pieces of the phantom material, one with and the other without glass powder, using piezoelectric crystals (one working as a transmitter and the other as a receiver) applied to the opposite sides of the test pieces. An electrical circuit and an oscilloscope connected to the crystals measured the time for a pulse of ultrasound to travel the distance between the crystals. The velocities of ultrasound within the phantoms were found to be 1470 m/s in the outer section with the glass powder and 1540 m/s in the inner section with iodixanol.

### Patients

Nine patients (5 men), aged 64 (range 51–82) years were selected consecutively from a larger, prospective study evaluating efficiency of coronary MSCT for the detection of coronary artery disease in patients referred routinely for coronary angiography due to known or suspected coronary artery or valvular diseases. In all the selected patients, 3-D echocardiography was performed in addition to MSCT. Both 3-D echocardiography and MSCT data were evaluated by independent trained interpreters. The study was approved by the local ethics committee of Karolinska University Hospital, Stockholm, Sweden.

### Simplified 3D-echocardiography

**Figure 1:**
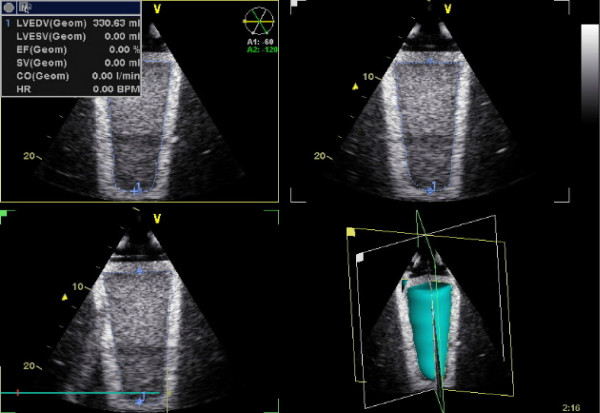
**Simplified 3D echocardiography**. Example of the tracing of the inner contour of the phantom in 3 planes positioned at 60° angle to each other and 3D reconstruction of phantom inner volume.

The echocardiographic study was performed using a GE Vingmed Vivid 7 equipment and V3 matrix transducer allowing simultaneous acquisition of image data in 3 different planes positioned at 60° angle to each other. In each of the 10 phantoms, the acquisition of echocardiographic data for the subsequent volume measurements was performed five times by placing the transducer close to the basal plane of the respective phantom submerged in water. In the patients, cineloops of 3 consecutive cardiac cycles were acquired at end-expiration from transthoracic apical window with a sampling frequency of 30 frames/s. The respective data sets were subsequently stored for offline analysis using a GE Echopac workstation and the Echopac software version BT06. In each of the three images generated simultaneously in three different planes by V3 Matrix transducer, the geometry and the area corresponding to LV cavity or to the inner section of the phantom (Fig. 1) was determined by manual border detection. Phantom volumes as well as human LV end-systolic and end-diastolic volumes were then estimated using Echopac software. LV ejection fraction was determined in human experiments as well.

### Multi-slice computed tomography

The MSCT examinations of both patients and phantoms were performed employing a 64-slice spiral computed tomography scanner (General Electric (GE) LightSpeed VCT, Milwaukee, Wisc., USA). The gantry rotation time was 0.35 seconds, while a collimation of 64 × 0.625 mm and a tube voltage of 120 kV was used. In the patients, dose modulation was used and the tube current was diminished during systole resulting in the effective current of approximately 240–640 mAs and effective dose of 20.0 mSv. All images (both in patients and phantoms) were acquired with slice thickness of 5 millimetres and spacing equal to 4 millimetres. A bolus injection of 85 ml iodixanol (Visipaque, 320 mgI/ml, GE Healthcare, Little Chalfont, UK) was given intravenously to the patients at a rate of 4–6 ml/sec followed by a flush with 50 ml of saline. Using retrospective electrocardiographic gating, reconstruction of images from 10 phases of the cardiac cycle was done for the patients.

### MSCT analysis of ventricular function

**Figure 2:**
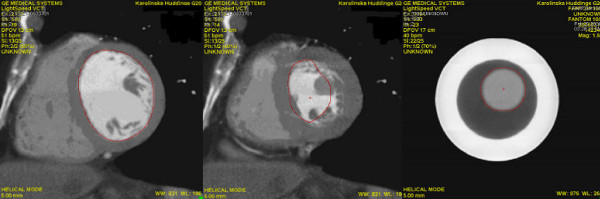
**Automatic contour detection by MSCT**. Examples of the automatic detection of the LV endocardial borders in short-axis CT images in diastole (0% of the R-R interval; left), and systole (40% of the R-R interval; middle) and the detection of inner contour of a phantom (right) using GE CardIQ Function Version 1.0.3 software.

The patient and phantom CT data sets were subsequently transferred to a dedicated workstation (Advantage Workstation 4.1, GE Medical Systems, Milwaukee, Wisc., USA) for further processing using GE CardIQ Function Version 1.0.3 software. Volumes of the phantoms and the left ventricles were evaluated using this semi-automated software. Data sets consisting of 5 mm thick axial images were loaded into the CardIQ software. For the patients, the images were reconstructed with retrospective ECG gating at 10% R-R interval increments throughout the cardiac cycle. The diastolic volume was determined at 0% of the R-R interval and the systolic volume at 40% of the R-R interval. Short-axis images were reconstructed and automatic contour detection of the inner-volume of the phantoms and the myocardial border in left ventricle was performed (Fig. 2). Minor manual corrections were made of the myocardial borders only, and not of the phantom borders. Short axis images not including any part of the left ventricle or phantom were manually excluded prior to the volume calculations. At the level of the mitral valve, short axis images usually show both part of the left ventricle and an area in the middle delineated by the mitral valve leaflets. Only images with the ventricle representing more than a half of the image area in this region were currently included. All manually selected images were processed further automatically and volumes were calculated. The papillary muscles were not automatically excluded from the calculated volume. The end-diastolic and end-systolic phases were selected and the volumes calculated. Five repeated volume measurements were performed in each phantom.

### Statistical analysis

Univariate relations between echocardiographic and MSCT-CardIQ measured LV volumes as well as their relation to the true phantom volumes were tested with standard regression analysis. Assessment of agreement between 3-D echocardiography and MSCT-CardIQ software in volume measurements was performed using the method of Bland and Altman [18]. Paired data were compared using the Student's *t*-test. The data are presented as mean ± SD unless otherwise stated.

## Results

### Phantom experiments

**Figure 3:**
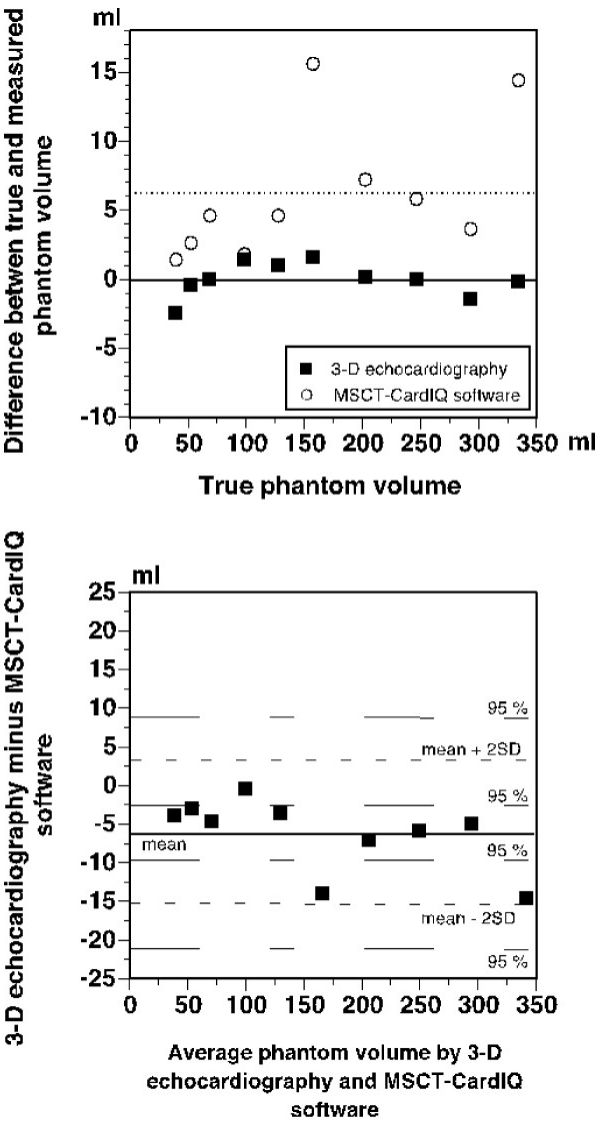
**Differences between 3D-echocardiographic and MSCT-derived phantom volumes**. Upper panel: Differences between true phantom volumes and the volumes measured by 3-D echocardiography and MSCT-CardIQ software. Mean differences for the respective methods are indicated. Lower panel: Bland-Altman plot of differences between phantom volumes measured by 3-D echocardiography and MSCT-CardIQ software against the average phantom volumes by the two methods. The mean difference and 95% confidence intervals for the mean difference ± 2SD are indicated.

The used MSCT CardIQ software provided fully adequate delineation of the inner section of the symmetric phantoms (Fig. 2, right) but failed to detect sharp irregularities of the inner contour of the asymmetric phantoms and manual correction was therefore necessary. The results of phantom volume measurements correlated strongly with true phantom volumes, both for 3-D echocardiography (r^2 ^= 1.00; y = 0.51 + 1.00x) and MSCT-CardIQ software (r^2 ^= 1.00; y = 0.17 + 1.03x). However, the results obtained with the two methods differed (p < 0.001). The true phantom volumes and the average volumes based on five volume measurements in each phantom with each method are presented in Table 1, whereas Figure 3 (upper panel) displays the differences between true phantom volumes and the volumes measured with 3-D echocardiography and MSCT-CardIQ software. As can be seen, the results obtained with MSCT-CardIQ software overestimated the true phantom volumes in all cases and were more scattered than the results of 3-D echocardiographic measurements, resulting in a mean difference of 6 ± 5 ml. The 95% confidence interval for the mean ± 2SD was +22 to +10 ml and +2 to -10 ml, respectively. On the other hand, the 3-D echocardiographic results were distributed more evenly and closely to the true volumes (mean difference 0 ± 1 ml) providing the true values in 2 cases and equal number of over- and underestimations for the rest of the phantoms. The 95% confidence intervals for the mean difference ± 2SD were +4 to -1 ml and +1 to -4 ml, respectively.

The poor agreement between 3-D echocardiography and MSCT-CardIQ software is further illustrated in Bland-Altman plot in Figure 3 (lower panel). The consistent overestimation of the phantom volumes by MSCT-CardIQ software resulted in a mean difference between the results obtained with the both methods amounting to -6 ± 5 ml. The limits of agreement defined as mean difference ± 2 SD were thus +4 ml and -16 ml, respectively. The 95% confidence interval for the upper limit of agreement was +9 to -3 ml and the corresponding confidence interval for the lower limit of agreement was -10 to -21 ml. This implies that the difference between the results of 3-D echocardiographic and MSCT-CardIQ software measurements may assume values ranging from +9 ml to -21 ml.

### Measurements in patients

Detection of the endocardial border and delineation of the LV cavity area in the patients by the MSCT-CardIQ software was less accurate than in the phantom experiments and required in most cases time consuming manual correction (Fig. 2, left and middle). A standard linear regression analysis revealed a moderate correlation between 3-D echocardiographic and MSCT-CardIQ software based volume measurements with r^2 ^= 0.62 (y = -10.99 + 1.19x) for the end-systolic, and r^2 ^= 0.59 (y = 32.39 + 0.91x) for the end-diastolic volume. Similar to what was observed in the phantom experiments, the results produced by the two tested methods (Table 2) differed significantly (p < 0.05), except for LV ejection fraction.

**Table 2:** Left ventricular volumes obtained in the same patient with 3-D echocardiography and MSCT-CardIQ software

		**Measured volumes (ml)**		**Ejection fraction (%)**
		**Endsystolic**	**Enddiastolic**	
	Patient 1	30	62	52
	2	31	89	65
	3	36	99	64
**3-D echo-**	4	40	116	66
**cardiography**	5	52	123	58
	6	61	158	61
	7	96	162	41
	8	104	178	42
	9	167	249	33

	Patient 1	40	145	72
	2	41	137	70
	3	36	116	69
**MSCT-CardIQ**	4	72	141	49
**software**	5	73	167	56
	6	61	164	63
	7	32	162	80
	8	91	170	46
	9	263	347	24

**Figure 4:**
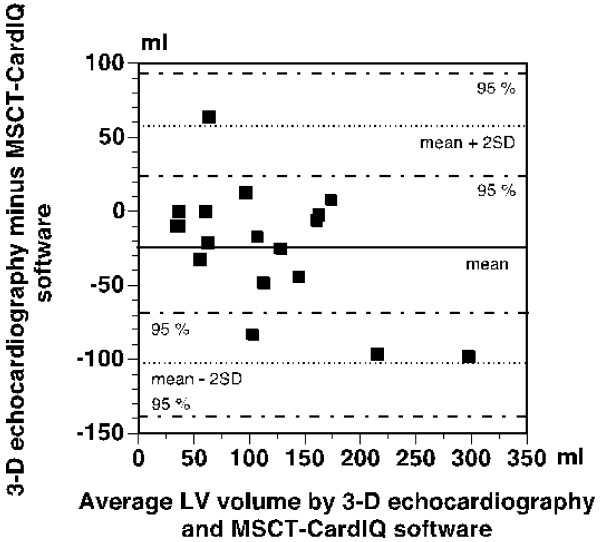
**Bland-Altman plot of differences between LV volumes measured by 3-D echocardiography and MSCT- seed growing software**. The mean difference and 95% confidence intervals for the mean difference ± 2SD are indicated. Filled symbols – differences in end-systolic volume, open symbols – differences in end-diastolic volume.

The agreement between the results of 3-D echocardiographic and MSCT-CardIQ software based measurements of LV volumes in the patients was clearly poorer than was the case with the measurements of phantom volumes as evidenced by Bland-Altman analysis presented in Figure 4. As can be seen from the figure, a mean difference between the measurements performed with the two methods was -23 ± 40 ml (-10 ± 42 ml for LV end-systolic and -35 ± 37 ml for LV end-diastolic volume measurements). The limits of agreement (mean ± 2SD) were +57 ml and -103 ml, respectively. The 95% confidence interval for the upper limit of agreement was +93 ml to +23 ml and the corresponding interval for the lower limit of agreement was -69 ml to -138 ml, thus indicating that the difference between the results obtained with the two tested methods may vary between +93 ml and -138 ml.

## Discussion

In the present study, the results of LV volume measurements by simplified 3-D echocardiography were compared with the results of 64-slice spiral computed tomography and the accuracy of the two methods was evaluated in two steps. First, both the methods were calibrated *in vitro *by comparison of the measured volumes with known volumes using phantoms, and second, the results of LV volume measurements with both the methods in patients were evaluated.

The phantoms used in the present experiments were especially designed to closely mimic the blood-myocardium border and to provide its equally good detection with both of the tested modalities. In order to fulfil these requirements, the speed of ultrasound wave travelling through the phantom should be in the range for which medical ultrasound equipment is calibrated for, i.e. around 1540 m/s, which is the speed of sound in most tissues imaged by echocardiography. The velocity of sound wave propagation through the currently employed phantoms was found to be 1470 m/s in the section with the glass powder, and 1540 m/s in the inner compartment of the phantom mimicking intracavital LV volume. The lower sound propagation velocity within the outer section of the phantoms resulted in a shift of depth coordinates for this section by +0.0475 mm per each mm. With the thickness of the phantom shell of approximately 8 mm, the total shift in its depth positioning would be about 0.4 mm and this would certainly introduce a systematic error if the total phantom volume was measured. However, since the velocity of ultrasound within the inner section of the phantoms was equal to the calibration velocity for the used equipment, imaging of the phantom "cavity" was not distorted and the currently performed calculations of the inner phantom volumes were therefore not biased.

The absolute value of the x-ray attenuation by the phantom structures was not as critical for the accuracy of the measurements as the velocity of sound wave propagation, but a proper automated border detection by the software required occurrence of a significant attenuation difference between the sections mimicking myocardium and LV cavity. In the present experiments, the chosen concentration of iodixanol in the inner section of the phantoms resulted in the same Hounsfield values as those obtained in routine diagnostic images and the delineation of this section was fully adequate in the case of the symmetric phantoms. The delineation of the "cavities" of the asymmetric phantoms was not entirely satisfactory and sharp contour irregularities of the inner section were not detected resulting in a tendency to overestimation of the phantom "cavity" volume that necessitated manual correction. These inadequacies were, however, not caused by insufficient attenuation differences but by software-dependent limitations to handle highly irregular borders.

The results of phantom volume measurements by simplified 3-D echocardiography showed very good agreement with the true phantom volumes resulting in mean difference between the respective data close to zero with a possible overestimation or underestimation of true volumes by 4 ml that is fully acceptable in clinical practice. On the other hand, the results produced by MSCT-CardIQ software were significantly more biased with a possibility of overestimation by 22 ml or underestimation by 10 ml. Despite the fact that there was a strong relationship between the true phantom volumes and the volumes measured with MSCT-CardIQ software, considerable discrepancies might occur between the true and measured values, and consequently, also between the results obtained with the both methods, thus limiting their interchangeable clinical use.

The poor agreement between the results of 3-D echocardiography and those generated by MSCT-CardIQ software was particularly striking when LV volume measurements in patients were compared. Several previous studies in which assessment of LV volumes with MSCT technique and 2-D echocardiography was evaluated produced results showing a good agreement between these two methods [4-7,19]. In addition, a good correlation was found between MSCT measurements, and between 3-D echocardiography and magnetic resonance imaging [2-4,8,9,11,14,17]. Even if the methodological details and the MSCT software used in the above-mentioned studies differed from the present approach, a similar good relationship with acceptable volume assessments could still be expected to exist between MSCT and 3-D echocardiography. However, the volume overestimation by MSCT-CardIQ software in relation to 3-D echocardiography was found to be considerable in clinical situation and the mean difference between the results obtained with two methods increased from moderate -6.2 ml in phantom experiments to -22.6 ml in patient measurements. At the same time the variation of the results increased considerably resulting in unacceptable limits of agreement ranging between +92.9 ml and -138.1 ml, thereby somewhat in disagreement with some of the above-mentioned earlier published data. On the other hand, the present results showing the larger volumes obtained by MSCT than those measured by 3-D echocardiography are in keeping with the results of recently published study of Sugeng et al. [17], in which MSCT was found to produce significant overestimation of LV volumes measured by magnetic resonance whereas real-time 3-D echocardiography compared more favourably in this respect.

When considering the results of 3-D echocardiographic and MSCT-CardIQ software based measurements of LV volumes, it has to be kept in mind that the methodological error may be greater in clinical setting than in *in vitro *phantom measurements. The simplified 3-D echocardiographic technique relies on a computation of LV volume by interpolation of manually traced endocardial border from three different 2-D planes. Consequently, any possible local changes in LV geometry between the traced planes will remain undetected, resulting in calculation error. On the other hand, the delineation of LV area contour with MSCT software may underestimate or overestimate the contribution of the contrast filled crevices and small cavities of the LV myocardial trabecular network. Multiplied by slice thickness, these possible area errors would inevitably result in erroneous LV volume estimations. In addition, the relatively low temporal resolution of the method and the nature of the used MSCT software calculating LV volume by integrating data from short-axis slices make the determination of volumes near to mitral annulus uncertain. However, the discrepancy between the 3-D echocardiographic and MSCT-CardIQ software generated LV volumes in the present study was substantial and suggests the existence of other colluding sources of errors as well. Since the MSCT software performed rather unsatisfactory on phantoms as well, it appears reasonable to believe that the observed considerable differences between the two methods might have been caused by additional, possibly MSCT algorithm dependent error. If so, the present results cast doubt on diagnostic applicability of the currently tested MSCT approach.

**Figure 5:**
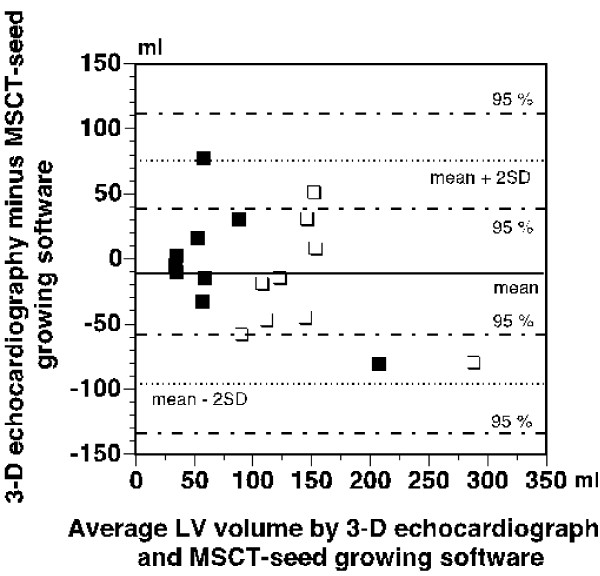
**Bland-Altman plot of differences between LV volumes measured by 3-D echocardiography and MSCT-CardIQ software**. The mean difference and 95% confidence intervals for the mean difference ± 2SD are indicated. Filled symbols – differences in end-systolic volume, open symbols – differences in end-diastolic volume.

As an additional comment to the present results, it is perhaps worthwhile to mention that after completion of this study, we also compared simplified 3-D echocardiography with another MSCT software (GE Software Ejection Fraction). This was a seed growing software that instead of outlining the endocardial border, worked by binding voxels with similar Hounsfield values assumed to originate from the same sort of tissue. Since the software did not work satisfactorily in phantoms, only measurements of LV volumes in patients were performed. The results are presented in Bland-Altman plot in Figure 5 and show that the agreement between this MSCT software and 3-D echocardiography was poor as well and clearly not better than that with MSCT-CardIQ software (mean difference ± SD equal to -11 ± 43 ml and limits of agreement 75 and -97 ml). The 95% confidence intervals for mean ± 2SD (112 to 39 ml and from -60 to -134) indicated that the difference between the methods might vary to the same considerable extent as for MSCT-CardIQ software.

In this context, it should be remembered that beside the currently reported results of volume measurements, there are other important factors that would favour the diagnostic use of 3-D echocardiography. For example, the effective radiation dose associated with the MSCT procedure employed in the present study was 20.0 mSv that is a dose equivalent to 1000 chest x-rays or nearly 7 years of background radiation [20]. This dose results in an extra risk of long-term cancer development of about 1/1000 exposed individuals and the procedure is at the same time about 3 times more expensive that conventional echocardiography [21]. On the other side, the 3-D echocardiographic approach, in addition to being radiation-free and more cost-effective, does not require the use of nephrotoxic contrast media, and is faster and easier to perform. These facts should be taken into consideration when adopting As Low As Reasonably Achievable (ALARA) philosophy when performing diagnostic cardiac imaging.

## Conclusion

The present results demonstrate that simplified 3-D echocardiography provides a reliable and significantly more accurate assessment of phantom volumes than MSCT-CardIQ software. The discrepancy between the results of both methods increase considerably when LV volume is measured and the limits of agreement are not acceptable for interchangeable diagnostic use of the both methods. Judging from its performance on phantoms, simplified 3-D echocardiography can be expected to provide most accurate LV volume assessments in clinical situations.

## Competing interests

The authors declare that they have no competing interests.

## Authors' contributions

MM constructed the phantoms, performed phantom volume measurements with 3-D echo and MSCT, and prepared the manuscript. RW performed the 3-D echo examination and LV volume calculation on patients. KC, JR and HMA performed MSCT examinations on both phantoms and patients. They performed volume calculations in patients with both software and participated in the manuscript preparation. JN performed statistical analysis and was responsible for the final layout of the manuscript. LÅB initiated and supervised the study and participated in the manuscript preparation. All authors read and approved the manuscript.
